# Paclitaxel-Loaded Folate-Targeted Albumin-Alginate Nanoparticles Crosslinked with Ethylenediamine. Synthesis and In Vitro Characterization

**DOI:** 10.3390/polym13132083

**Published:** 2021-06-24

**Authors:** Ana María Martínez-Relimpio, Marta Benito, Elena Pérez-Izquierdo, César Teijón, Rosa María Olmo, María Dolores Blanco

**Affiliations:** 1 Facultad de Ciencias Experimentales, Universidad Francisco de Vitoria, Pozuelo de Alarcón, 28223 Madrid, Spain; am.martinez.prof@ufv.es; 2 Fundación San Juan de Dios, Centro de Ciencias de la Salud San Rafael, Universidad de Nebrija, Paseo de La Habana, 70, 28036 Madrid, Spain; mbenito@nebrija.es; 3 Department of Health Sciences, Faculty of Biomedical and Health Sciences, Universidad Europea de Madrid, Urbanización El Bosque, Calle Tajo, s/n, 28670 Villaviciosa de Odón, Spain; 4 Nursing Department, Faculty of Nursing, Physiotherapy and Podiatry, Universidad Complutense de Madrid, 28040 Madrid, Spain; cteijon@ucm.es; 5 Department of Biochemistry and Molecular Biology, Faculty of Medicine, Universidad Complutense de Madrid, 28040 Madrid, Spain; rmolmo@med.ucm.es (R.M.O.); mdblanco@med.ucm.es (M.D.B.)

**Keywords:** folate-targeted nanoparticles, BSA/alginate nanocarriers, paclitaxel, cellular uptake, cell viability

## Abstract

Among the different ways to reduce the secondary effects of antineoplastic drugs in cancer treatment, the use of nanoparticles has demonstrated good results due to the protection of the drug and the possibility of releasing compounds to a specific therapeutic target. The α-isoform of the folate receptor (FR) is overexpressed on a significant number of human cancers; therefore, folate-targeted crosslinked nanoparticles based on BSA and alginate mixtures and loaded with paclitaxel (PTX) have been prepared to maximize the proven antineoplastic activity of the drug against solid tumors. Nanometric-range-sized particles (169 ± 28 nm–296 ± 57 nm), with negative Z-potential values (between −0.12 ± 0.04 and −94.1± 0.4), were synthesized, and the loaded PTX (2.63 ± 0.19–3.56 ±0.13 µg PTX/mg Np) was sustainably released for 23 and 27 h. Three cell lines (MCF-7, MDA-MB-231 and HeLa) were selected to test the efficacy of the folate-targeted PTX-loaded BSA/ALG nanocarriers. The presence of FR on the cell membrane led to a significantly larger uptake of BSA/ALG–Fol nanoparticles compared with the equivalent nanoparticles without folic acid on their surface. The cell viability results demonstrated a cytocompatibility of unloaded nanoparticle–Fol and a gradual decrease in cell viability after treatment with PTX-loaded nanoparticle–Fol due to the sustainable PTX release.

## 1. Introduction

Targeted treatments and personalized medicine are two of the axes of current oncology. They refer to the design of drugs that respond specifically to some genetic characteristics of each patient’s cancer. More efficiency is pursued with fewer adverse effects, adapting the procedures to the characteristics of each patient. Numerous studies have focused on the development of targeted drug-delivery systems [1] for cancer treatment in order to reduce side effects due to the unspecific effect of anticancer drugs on healthy cells. Nanocarriers based on natural polymers have the advantage of being a priori biocompatible and also biodegradable. Among biological polymers, polysaccharides have been used for therapeutic delivery systems [2] due to their non-toxic and non-reactogenic properties, as well as their physicochemical properties that allow chemical modifications and, therefore, an easy preparation of nanocarriers. Their reactive groups, such as hydroxyl, carboxyl or amine, can attach different molecules and so introduce new physicochemical characteristics [3]. Anionic polysaccharides have an important role in the preparation of drug delivery systems [4]; they have extensively contributed to the development of several types of nanocarriers for cancer treatment and diagnosis. Among anionic polysaccharides, alginate, whose structure is based on a backbone of [1,2,3,4] linked β-D-mannuronic acid (M units) and α-L-guluronic acid (G units), have been used for preparing different nanocarriers for biomedical applications in the last years [5]. Alginate has been declared safe by the FDA [6] for application in humans as a dental impression material or wound dressing, and their hydrogels are the most assayed material for bone tissue engineering and bioprinting since they provide an appropriate niche for cell loading [7]. On the other hand, there is significant interest in developing anticancer drug carriers based on serum albumin [8] due to the therapeutic efficacy of Abraxane**^®^**, an albumin-bound form of paclitaxel, that rapidly dissociates in serum, losing some benefits of nanoformulations. Some studies have indicated that Abraxane^®^ induced the overexpression of P-glycoprotein and did not allow it to overcome the common small molecule drug resistance problem mediated by P-gp [9]; in addition, when crosslinked and non-crosslinked PTX-loaded albumin nanoparticles were compared, differences in pharmacokinetics were observed, due to their different physiological ways of delivering the drug to the tumor [10]. In an attempt to improve the characteristics of nanocarriers, numerous studies have focused on the synthesis of nanosystems formed by proteins and polysaccharides; in this way, core–shell microcapsules based on BSA gel with a polyelectrolyte complex multilayer shell of hyaluronic acid and chitosan, encapsulating Sorafenib, have been designed for hepatocellular cancer therapy [11]. Ionic crosslinked nanoparticles based on chitosan and BSA were also developed as carriers for doxorubicin, showing their biocompatibility after intravenous administration in a *Wistar* rat model [12]. Nanocarriers can reach the internal part of solid tumors, taking advantage of their angiogenesis, but the uptake of these nanosystems improves significantly if they are targeted to overexpressed receptors on the surface of cancer cells. Among those receptors [13], the axis folic acid/folate receptor is one of the most important ligand–receptor interactions used to target cancer. Folic acid reaches normal tissues through two carriers, the reduced folate carrier (RFC) and the proton-coupled folate transporter, but either of them are able to bind folate conjugates (such as folate drugs or folate nanocarriers). Folate-bounded nanocarriers are internalized by the membrane-bound folate receptor (FR), which has a high affinity for folate (Kd ∼ 0.1–1 nmol/L) although is expressed on few cell types. The FR-α isoform is overexpressed on 40% of human cancers (such as ovary, kidney, breast and liver tumors) where it is completely accessible to FA-bounded systems, unlike what happens in healthy cells, where the FR-α isoform is detected in the apical membrane of some epithelial cells, where folate conjugates do not have access. This fact makes folate-targeted nanosystems [14,15] very selective for cancer cells.

In the present study different compositions of folate-targeted nanoparticles based on BSA and alginate have been prepared for use as an active targeting strategy for the delivery of paclitaxel (PTX). Thus, the challenge of this study was the preparation of stable nanoparticles capable of specifically delivering that hydrophobic drug in a prolonged time in the environment of many solid tumors, avoiding the hypersensitivity, nephrotoxicity and cardiotoxicity reactions induced by the conventional use of Cremophor^®^EL [16] and drug resistance problem mediated by the aforementioned P-gp [9]. These nanoparticles were in vitro characterized to evaluate their application in targeted tumor therapy.

## 2. Materials and Methods

### 2.1. Materials

Folic acid (FA), alginic acid sodium salt (ALG; viscosity 15.00–20.00 cps, 1% H_2_O), trypsin from bovine pancreas (13,000 units/mg solid), gentamicin (50 µg/mL), dimethyl sulfoxide (DMSO), methylthiazoletetrazolium (MTT), 6-coumarin, ethylenediamine (ED; 98%), N-hydroxysuccinimide (NHS), N,N’-dicyclohexylcarbodiimide (DCC) were purchased from Sigma-Aldrich (Barcelona, Spain). Sodium hydroxide (NaOH), hydrochloric acid (HCl; 35%), trichloroacetic acid, ethanol absolute, ethylenediaminetetraacetic acid (EDTA), Tween^®^80, triethylamine, anhydrous di-sodium hydrogen phosphate (Na_2_HPO4), di-hydrogen potassium phosphate (KH_2_PO4), diethylether, dichloromethane (DCM) and acetonitrile (HPLC-analysis grade) were supplied by Panreac (Madrid, Spain). Bovine serum albumin (BSA, Fraction V), sodium chloride (NaCl) and disodium hydrogen phosphate dehydrated were purchased from Merck (Barcelona, Spain). Fetal bovine serum (FBS), penicillin (50 U/mL), streptomycin (50 µg/mL) and 0.05% trypsin/0.53 mM EDTA were purchased from Invitrogen Life Technologies, Grand Island, NY. Dubecco’s Modified Eagle Medium was purchased from Lonza Bioscience (Bornem, Belgium). Paclitaxel (Taxol) was supplied by Tocris Bioscience (Mw 889.95 > 99%). All water used in the assays was Millipore Milli Q grade.

### 2.2. Preparation of BSA/ALG Nanoparticles

Folate-targeted nanoparticles (Nps–Fol) based on bovine serum albumin (BSA) and alginate (ALG) mixtures were prepared and stabilized by amide bonds using ethylenediamine. BSA 5% (*w*/*v*) and ALG 1% (*w*/*v*) aqueous solutions were prepared in the presence of EDAC 50 mM (Sigma-Aldrich). Subsequently, several blends of BSA/ALG were obtained by mixing different volumes of the solutions prepared previously (Supplementary data Table S1). Ethylenediamine (ED) was added to each blend according to 2:1 ratio (polymer: ED, *w*/*w*) under intense stirring conditions (magnetic stirrer), and pH was then adjusted to 3 with HCl to attain amide bond formation. Stirring was maintained for 2 h and blends were finally centrifuged (10,000 rpm, 5 min; Sigma SM202 centrifuge). Pellets were freeze dried (Heto PowerDry LL1500 Freeze Dryer, Thermo Electro Corporation, Waltham, MA, USA) for 24 h at −110 °C.

The conjugation of folate to nanoparticles was carried out by a carbodimiide reaction with NHS–folate. The N-Hydroxysuccinimide ester of folic acid (NHS–folate) was prepared according to the method developed by Lee and co-workers [17]. Folic acid (2.5 g) was dissolved in 50 mL of dry dimethyl sulfoxide (DMSO) plus 1.25 mL of triethylamine and reacted with NHS (1.3 g) in the presence of dicyclohexylcarbodiimide (2.35 g) overnight at room temperature. The byproduct, dicyclohexylurea, was removed by filtration. The DMSO solution was then freeze-dried, and NHS–folate was precipitated in diethylether. The final product, NHS–folate, was washed several times with anhydrous ether, dried under vacuum, and yielded a yellow powder.

The conjugation of folate to nanoparticles was carried out following the method developed by Zhang and co-workers [18]: NHS–folate (50 mg/mL) was dissolved in 3 mL DMSO and added slowly to 6 mL of the stirred nanoparticle suspension (10 mg NP/mL, pH adjusted to 10 using 1 M carbonate/bicarbonate buffer). After stirring for 3 h at room temperature, the reaction mixture was centrifuged (10,000 rpm, 10 min) in order to separate the folate conjugated nanoparticles from unreacted folic acid and other byproducts and then washed with DMSO (10,000 rpm, 10 min). Finally, folate-conjugated nanoparticles (30BSA/70ALG–Fol, 40BSA/60ALG–Fol; 50BSA/50ALG–Fol) were freeze dried for 24 h at −110 °C.

### 2.3. Characterization of Nanoparticles

#### 2.3.1. Composition of BSA/AlG Nanoparticles

Composition of nanoparticles was determined according to the method developed by Martinez and co-workers [19]. The composition of BSA/ALG nanoparticles was studied measuring the concentration of BSA and ALG in the supernatant obtained after the centrifugation of the samples in the synthesis process. The difference between the initial concentration of each polymer and their concentration in the supernatant was determined, to reveal the proportion of each polymer in nanoparticle formation. Concentration of alginate in the supernatant was determined by measuring the absorbance of the colored products obtained after a hydrolysis reaction of alginate. The hydrolysis of alginate was carried out in extreme conditions (10 N HCl, 5 h, 100 °C) and, after this reaction, samples were neutralized with 10 N NaOH and cooled. Absorbance of the samples was measured at 277.6 nm. A standard curve of alginate (0.5–4 mg/mL) was prepared. Concentration of BSA in the supernatant was determined using Bradford’s method [20]. The absorbance of blue-colored products of this reaction could be measured at 595 nm. To avoid interactions between alginate and Bradford reagent, the BSA in the supernatant was precipitated with 2M trichloroacetic acid (TCA). After the precipitation process, samples were centrifuged and the supernatant was removed. The pellet was suspended in 1 mL 6M urea (urea solution in 2 N HCl). A standard curve of BSA (0.125–0.5 mg/mL) was prepared.

#### 2.3.2. Thermogravimetric Analysis (TGA)

TGA curves of BSA/ALG nanoparticles, as well as raw materials, were obtained using a Mettler Toledo thermal analyzer (TGA-SDTA 851^®^, Switzerland). Samples (3 mg) were placed onto the balance, the temperature increased from 25 to 600 °C at 10 °C min^−1^ in a nitrogen atmosphere (nitrogen flow rate of 60 cm^3^ min^−1^) and the mass continuously recorded as a function of temperature.

#### 2.3.3. Determination of Folate-Conjugated to BSA/ALG Nanoparticles

The amount of folate conjugated to the amine groups of albumin was determined by spectrophotometric analysis [18]. A quantity of 2 mg folate-conjugated nanoparticles (Nps–Fol) were hydrolysed by trypsin (0.05 mg/mg NP) with stirring at 37 °C for 2 h. After the digestion process, the quantification of folate-conjugation was performed by spectrophotometric measurement of its absorbance at 358 nm (folic acid ε = 8643.5 M^−1^ cm^−1^).

#### 2.3.4. Morphology, Size and Z-Potential of Nanoparticles

The morphology and size of Nps and Nps–Fol were studied by Transmission Electron Microscopy (TEM) (JOEL JEM 1010 microscope from ICTS Centro Nacional de Microscopía Electrónica, UCM). Size and zeta potential measurements of Nps and Nps–Fol were performed in deionized distilled water using a backscattered quasi-elastic light scattering device (Zetatrac NPA). Data were analyzed with Microtrac Flex Software.

#### 2.3.5. Preparation of PTX–Folate-Conjugated Nanoparticles

The paclitaxel (PTX) load into Nps–Fol was carried out as follows: a suspension of 30 mg of NPs–Fol in 1 mL of the solution 1 mg PTX/mL ethanol was prepared and incubated for 22 h using a circular rotary stirrer in darkness at room temperature. Finally, the suspension was centrifuged (12,000 rpm, 15 min), the pellet was washed with water and nanoparticles were freeze-dried.

#### 2.3.6. Estimation of PTX Content

Two different methods were carried out in order to determine the amount of PTX loaded into Nps–Fol. One of the methods consisted of a direct extraction of the drug from PTX-loaded Nps–Fol using ethanol: 3 mg of PTX-loaded Nps–Fol were suspended in 1 mL of ethanol under stirring conditions for 5 h. After this time, the suspension was centrifuged (13,000 rpm, 5 min) and the supernatant was collected. The samples were analyzed by HPLC. The other method consisted of an enzymatic digestion of PTX-loaded Nps–Fol with trypsin and a subsequent extraction of the drug with an organic solvent: 2 mg of PTX-loaded nanoparticles was suspended in 2 mL of phosphate-buffered saline (PBS, pH 7.4), and 50 µg of trypsin was added to the solution. The digestion process was performed under orbital stirring (100 rpm) at 37 °C for 24 h. After this time, PTX was extracted from PBS solution by the addition of 4 mL of dichloromethane. The tubes were mixed in a vortex mixer for 10 min and then centrifuged for 10 min at 10,000 rpm. The organic phase was transferred to flask vials and evaporated to dryness. The resulting residue was reconstituted in ethanol and then quantified by the HPLC technique.

The concentration of PTX was determined by high-performance liquid chromatography (HPLC) using a Spectra Physics HPLC system, Spectra 100 UV–Vis detector and a SP8800 pump. The mobile phase consisted of acetonitrile/water 60:40 (*v/v*) and was pumped through 25 mm × 4.6 mm RP-Spherisorb ODS2 C18 column (5 mm particle size, Waters) at a flow rate of 1 mL/min. The output at 227 nm was monitored and the peak area of each sample generated by ChromQuestTM software. The calibration curve was generated using PTX solutions between 0.1 and 25 µg/mL in acetonitrile/water (60:40, *v/v*), prepared from a PTX solution in ethanol, and a good linear correlation (r^2^ = 0.99) was obtained. All quantifications were performed in triplicate.

#### 2.3.7. In Vitro Drug Release Studies

The release of PTX from Nps–Fol was carried out in centrifuge tubes. Drug-loaded Nps–Fol (3 mg) was suspended in PBS (4 mL pH 7.4, 37 °C) containing 0.1% (*w/v*) Tween 80^®^ [21,22]. Release studies were carried out in the dark with orbital shaking (100 rpm; Ecotron INFORS HT, Switzerland) for 96 h. Sink conditions were maintained during drug release experiments and all experimental conditions were performed in triplicate. At predetermined time intervals, the solutions were centrifuged at 10,000 rpm for 1 min and the supernatants collected for PTX concentration analysis. The Nps–Fol samples were dispersed in fresh PBS and placed back on the shaker. The PTX was extracted from the supernatants by DCM (1 mL) by stirring using a vortex mixer for 10 min at room temperature. The DCM layer collected was evaporated at room temperature for 24 h, and the dried PTX was dissolved in acetonitrile/water (1 mL; 60:40 (*v/v*)). The percentage of PTX released into the supernatant was determined by HPLC.

In order to analyze the drug delivery mechanism used by the NPs–Fol, the in vitro drug release data were fitted to various kinetic models. Higuchi, Korsemeyer–Peppas, zero-order and first-order models were selected for this study, applying the following set of equations [23]:

Higuchi model

M_t_ = K_H_ √t

Korsemeyer–Peppas

M_t_/M_∞_ = Kt^n^

Zero-order kinetic model

M_o_ − M_t_ = K_o_t

First-order kinetic model

M_t_/M_∞_ = 1 − e^−Kt^

where M_o_, M_t_ and M_∞_ corresponded to the drug amount at time zero, at a particular time and at an infinite time, respectively. The K terms referred to the release kinetic constants obtained from the linear curves of simple regression analysis.

#### 2.3.8. Cell Culture Studies

Human breast adenocarcinoma (MCF7, MDA-MB-231) and human cervical cancer HeLa cell lines were obtained from ECACC (Sigma-Aldrich).These cells were chosen due to their expression of folate receptors (FRs) [24,25,26]. Cells were maintained in DMEM + GlutaMax-I supplemented with 10% heat inactivated fetal bovine serum, penicillin (50 U/mL), streptomycin (50 µg/mL) (Invitrogen Life Technologies, Grand Island, NY, USA) and gentamicin (50 µg/mL) (Sigma–Aldrich Company, Gillingham, UK) in a humidified incubator at 37 °C and 5% CO2 atmosphere (HERA cell, Sorval Heraeus, Kendro Laboratory Products GmbH, Hanau, Germany). Cells were plated in a 75 cm^2^ flask (Sarstedt Ag and Co., Barcelona, Spain) and were passaged when they reached 90% confluence by gentle trypsinization (0.05% trypsin/0.53 mM EDTA; Invitrogen Life Technologies).

##### Cellular Uptake of Nanoparticles

Nps and Nps–Fol (10 mg) were loaded with 6-coumarin (98%, Sigma-Aldrich, Madrid, Spain) by immersion in 1 mL of a 1 mg/mL 6-coumarin solution (dissolved in ethanol) for 24 h at room temperature in the dark and under orbital shaking. Then, the suspension was centrifuged (7000 rpm, 5 min), and the 6-coumarin-loaded NPs were washed twice with ethanol and freeze-dried for 24 h at −110 °C

Cellular uptake of Nps and Nps–Fol was investigated in a monolayer of MCF7, MDA-MB-231 and HeLa cells. Cells were seeded in 96 well flat-bottom plates at 10,000 cells/well for MCF7 and MDA-MB-231, and 5000 cells/well for HeLa. After 24 h, the medium was replaced with 100 µL of 1% FBS medium containing 6-coumarin-loaded Nps or Np–Fol and the plates were incubated in the 5% CO2 incubator at 37 °C for 2, 4 and 24 h. The concentration of nanoparticles was the equivalent to obtain a concentration of 1 µM of drug, considering the PTX load of each nanoparticle composition. Thus, the concentration of 6-coumarin-loaded Nps and Nps–Fol was 0.24 mg/mL (composition 50BSA/50ALG) and 0.325 mg/mL (composition 30BSA/70ALG) respectively. As a positive control, cells were grown with 0.2 µg/mL of 6-coumarin in FBS medium, in the absence of nanoparticles. At each time interval, cells were washed twice with PBS to remove any uninternalized nanoparticles and lysed with 100 µL lysis reagent (PBS with 2% SDS *v/v* and 50 mM EDTA). Internalization of nanoparticles was evaluated by fluorimetry at 488 nm, using a spectrophotometer (Varioskan, Thermo FisherScientific, Barcelona, Spain) [27]. Calibration curves (fluorescence versus concentration) were prepared with 6-coumarin (1 ng/mL^−1^ µg/mL) dissolved in the lysis medium in order to calculate the amount of 6-coumarin incorporated into nanoparticles. All experimental conditions were performed in quintuplicate.

##### Fluorescence Microscopy

Cellular uptake of Nps and Nps–Fol with 6-coumarin was observed by fluorescence microscopy. Cells were seeded in 24 well flat-bottom plates at 40,000 cells/well for MCF-7 and MDA-MB-231, and 20,000 cells/well for HeLa. After 24 h, the medium was replaced with 500 µL of 1% FBS medium containing 6-coumarin-loaded Nps or Np–Fol and the plates were incubated in the 5% CO_2_ incubator at 37 °C for 4 h. Then, cells were washed twice with PBS to remove any uninternalized nanoparticles. Localization of Nps–Fol with 6-coumarin was examined by light fluorescence microscopy (Leica DMIL microscope, Leica Microsystems, Balgach, Switzerland). Cells were photographed with a Leica DFC 300FX digital camera and Leica Application Suite software was used for processing the pictures (Leica Microsystems Switzerland).

##### Cell Viability

Cell viability was evaluated by using the MTT method. All experimental conditions were performed in quintuplicate. Each experiment was carried out in triplicate.

In preliminary experiments, increasing concentrations of PTX from 0.1 to 500 nM were tested in culture. The IC50 of PTX was determined in the three cell lines tested. According to that, the selected concentrations of PTX in cell viability studies were 2.5 and 7.5 nM for HeLa and MCF7 cell lines, and 7.5 and 30 nM for MDA-MB-231 cells.

Cells were seeded in 96-well flat-bottom plates at 5000 cells/well in the case of MCF7 and MDA-MB-231, and at 2500 cells/well in the case of HeLa. After 24 h, the medium was replaced with 100 µL medium with 1% FBS containing Nps–Fol, PTX-loaded Nps–Fol or the drug in solution. The amount of Nps–Fol was the needed to obtain a final concentration of 30, 7.5 and 2.5 nM of PTX, considering the PTX load of each nanoparticle composition; for this purpose, a nanoparticle suspension of 0.24 mg/mL of 50BSA/50ALG and 0.325 mg/mL of 30BSA/70ALG Nps–Fol, (equivalent to 1000 nM PTX) was used. After 1, 3 and 6 days, 10 µL of MTT solution (5 mg/mL) were added to each well. After 2 h of incubation at 37 °C, 5% CO2, each well was replaced with 100 µL DMSO [24]. The cell viability was determined by measuring the absorbance at 570 nm using a spectrophotometer (Varioskan, Thermo Fisher Scientific, Barcelona, Spain). Results are expressed as the percentage survival in relation to untreated control cells.

### 2.4. Statistical Analysis

Statistical comparisons were performed with the unpaired Student’s *t*-test. A value of *p* < 0.05 was considered significant.

## 3. Results

### 3.1. Preparation of BSA/ALG Nanoparticles

#### 3.1.1. Composition of Nanoparticles

Final nanoparticle composition was determined by the concentration of BSA and ALG in the supernatant collected after centrifugation, in the synthesis process. Thus, the contribution of each polymer within the final formulation of nanoparticles was calculated by the difference between the initial concentration and the final concentration after the synthesis protocol (Table 1). The BSA/ALG ratio was calculated considering the concentration of each polymer incorporated into nanoparticles. These results demonstrated that not all the BSA nor ALG added in the initial solution of synthesis was incorporated in the final nanoparticles. Surprisingly, an increased proportion of BSA (*w/w*) in the initial composition was not translated into a higher incorporation of BSA into Nps.

#### 3.1.2. Thermogravimetric Analysis (TGA)

The thermal stability of raw materials and BSA/ALG nanoparticles was studied. The TGA first derivative of the materials is plotted in Figure S1 (Supplementary Materials). The Nps showed a thermal profile different from raw materials: Nps that included more BSA in their composition (30BSA/70ALG, ratio 1:1) showed a two-step degradation process: at 217 °C (close to the degradation peak of ALG) and 317 °C (close to the degradation peak of BSA). However, Nps with more ALG in their composition (40BSA/60ALG, ratio 0.25:1; 50BSA/50ALG, ratio 0.5:1) showed only one degradation peak at 212 °C (close to the degradation peak of ALG). These differences in thermal behavior between raw materials and the Nps would indicate the chemical interaction between both polymers in the Nps and would confirm the different ratio of polymers incorporated in each Np composition. Additionally, all Np compositions showed a mass loss above 200 °C which indicated that Nps were stable to use at routine work temperatures.

#### 3.1.3. Determination of Folate Conjugated to Nanoparticles

The amount of folate conjugated to Nps–Fol was spectrophotometrically evaluated after the hydrolysis of nanoparticles by trypsin. Values of 30 ± 2, 9 ± 2 and 26 ± 2 µmol Fol/g Nps were respectively obtained from 30BSA/70ALG–Fol, 40BSA/60ALG–Fol and 50BSA/50ALG–Fol. Statistically significant differences did not exist between 30BSA/70ALG–Fol and 50BSA/50ALG–Fol nanoparticles (*p* = 0.07) (data not shown); however, significant differences were observed between 30BSA/70ALG–Fol and 40BSA/60ALG–Fol (*p* = 0.0002) and also between 50BSA/50ALG–Fol and 40BSA/60ALG–Fol (*p* = 0.0004). Therefore, no correlation between the amount of BSA in the nanoparticles and the extent of folate on their surface was established.

According to these results, the compositions 30BSA/70ALG–Fol and 50BSA/50ALG–Fol were finally chosen for further studies due to their greater content of incorporated folate.

### 3.2. Characterization of Folate-Conjugated BSA/ALG Nanoparticles

#### 3.2.1. Morphology, Size and Z-Potential of Nanoparticles

**Figure 1:**
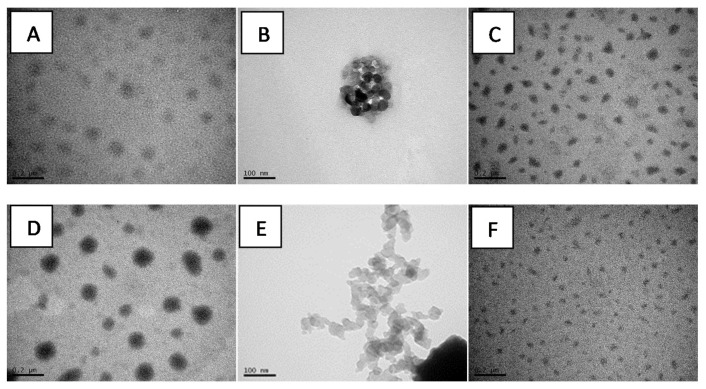
TEM micrographs of 30BSA/70ALG (**A**), 30BSA/70ALG–Fol (**B**), PTX-loaded 30BSA/70ALG–Fol (**C**), 50BSA/50ALG (**D**), 50BSA/50ALG–Fol (**E**) and PTX-loaded 50BSA/50ALG–Fol (**F**) nanoparticles.

The morphology of 30BSA/70ALG and 50BSA/50ALG Nps was analyzed by TEM. Micrographs showed nanometric-range sized particles (<100 nm), with a spherical appearance. This morphology was maintained after folate conjugation (Figure 1).

These nanoparticles were also analyzed using quasi-elastic light scattering to characterize them in terms of mean size and surface charge (Table 2). The results showed that all synthesized systems had a nanometric size in the range of 169 ± 28 nm and 296 ± 57 nm and a polydispersity index between 1.2 ± 0.4 and 1.8 ± 0.4. Moreover, an increase in size was observed after the incorporation of folate and the loading of paclitaxel in both nanoparticle compositions. However, these differences in size were not statistically significant. Differences in nanoparticle size among TEM photographs and DLS data can be attributed to the different characteristics of these techniques. In this way, DLS measures are realized in a water solution without stirring, and nanoparticles tend to establish interactions and aggregate, increasing the observed size.

Results of Z-potential analysis (Table 2) showed a negative charge at the surface of all the tested compositions, like the results obtained in similar studies [28]. The negative value of this parameter was increased after the conjugation of folic acid and the incorporation of PTX in both formulations, those differences were statistically significant.

#### 3.2.2. Estimation of PTX Content in Folate-Conjugate Nanoparticles

The estimation of PTX content in each formulation was determined by two methods: extraction with ethanol and enzymatic degradation using trypsin (Table 3). Independently of the method, results showed statistically significant differences (*p* < 0.05) in drug content comparing both compositions, showing a higher PTX load in 50BSA/50ALG–Fol nanoparticles.

#### 3.2.3. PTX Release from Folate-Conjugated Nanoparticles (Nps–Fol)

**Figure 2:**
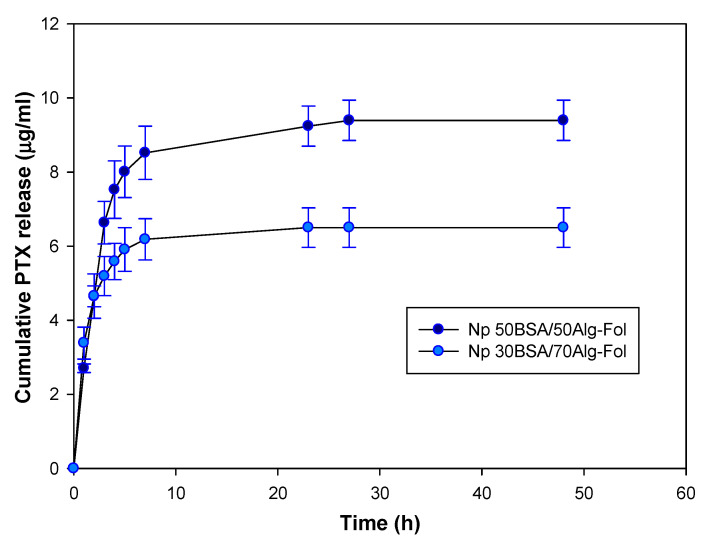
Cumulative release of PTX from BSA/ALG–Fol nanoparticles.

The cumulative release curve of PTX is represented in Figure 2. The maximum PTX release (82% and 88% of drug load) was achieved at 23 and 27 h from 30BSA/70ALG–Fol and 50BSA/50ALG–Fol, respectively. Drug release in the presence of Tween 80^®^ occurred quickly in the first 4 h (Table 4), followed by a slower release rate in both types of nanosystem. In both stages of drug delivery, the release of PTX was faster from the 50BSA/50ALG–Fol composition.

The PTX release was also evaluated in a Tween 80^®^ free medium at 74 h. In this experimental condition, only 16% and 23% of the PTX load was released from 50BSA/50ALG–Fol and 30BSA/70ALG–Fol, respectively.

### 3.3. In Vitro Evaluation of Folate-Conjugated Nanoparticles in Tumour Cell Lines

#### 3.3.1. Cellular Uptake of Nanoparticles

**Figure 3:**
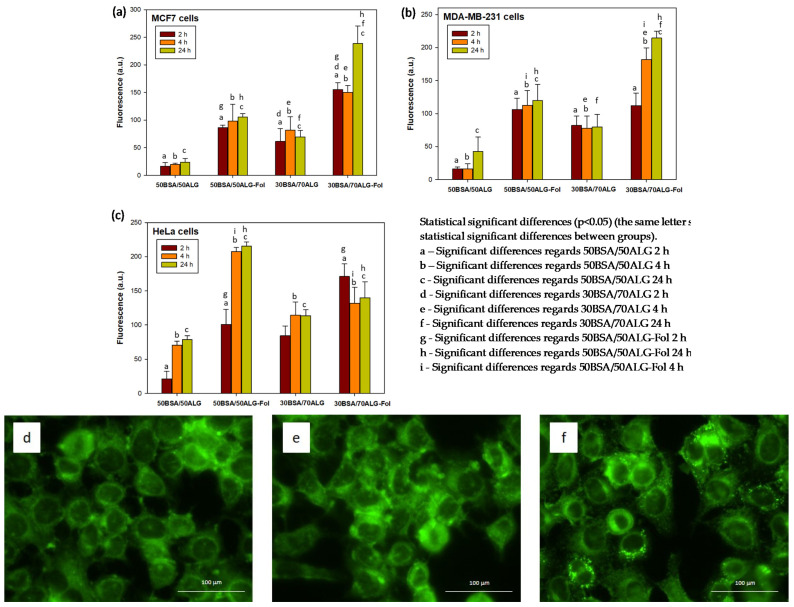
Quantitative comparison of coumarin-loaded internalized nanoparticles in (**a**) MCF-7, (**b**) MDA-MB-231 and (**c**) HeLa cells. Fluorescence microscopy images of coumarin-loaded internalized nanoparticles 50BSA/50ALG–Fol into MCF-7 (**d**), MDA-MB-231 (**e**) and HeLa (**f**) after 4 h of incubation.

To evaluate the uptake of the synthesized nanosystems by cells, three different human tumor cell lines, MCF-7, MDA-MB-231 and HeLa, were selected. BSA/ALG–Fol nanoparticles and BSA/ALG nanoparticles were loaded with a fluorescent marker (6-coumarin) and added to cells in order to study their cellular uptake. The fluorescent signal into cells was quantified after 2, 4 and 24 h of incubation. Results are shown in Figure 3. As results showed, either targeted or non-targeted systems were internalized in cells. However, folate-conjugated nanoparticles enhanced their uptake in the three cell lines. The 50BSA/50ALG nanoparticles were less internalized than 30BSA/70ALG.

Besides the quantitative assay, the internalization of these systems was easily observed due to the green fluorescence of coumarin attached to the particles. Figure 3 shows the uptake when they were functionalized with folate (BSA50/50ALG–Fol) for 4 h, as an example of the images obtained.

#### 3.3.2. Cell Viability

Tumor cell cytotoxicity induced by different concentrations of unloaded and PTX-loaded BSA/ALG–Fol nanoparticles was determined in MCF7, MDA-MB-231 and HeLa cells. IC50 values of PTX in solution were 7.32 nM, 7.71 nM and 38 nM for HeLa, MCF7 and MDA-MB-231 cells, respectively, after 24 h of incubation. According to these results, two concentrations of PTX (2.5 and 7.5 nM for the HeLa and MCF7 cell lines, and 7.5 and 30 nM for the MDA-MB-231 cell line) were selected to perform cell viability studies. Cell viability studies in the presence of unloaded and PTX-loaded 50BSA/50ALG–Fol and 30BSA/70ALG–Fol nanoparticles were carried out over one, three and six days using the equivalent concentration to 2.5 and 7.5 nM PTX for HeLa and MCF7 cell lines, and 7.5 and 30 nM PTX for the MDA-MB-231 cell line when the drug was administered by nanoparticles.

Both unloaded nanoparticles (50BSA/50ALG–Fol and 30BSA/70ALG–Fol) were cytocompatible and no significant decrease in viability (cell survival between 91% ± 6% and 89% ± 3% for MCF7; 83% ± 5% and 84% ± 13% for MDA-MB-231; 100% ± 3% and 100% ± 12% for HeLa) was observed for the three tested cell lines at longer times of exposure.

**Figure 4:**
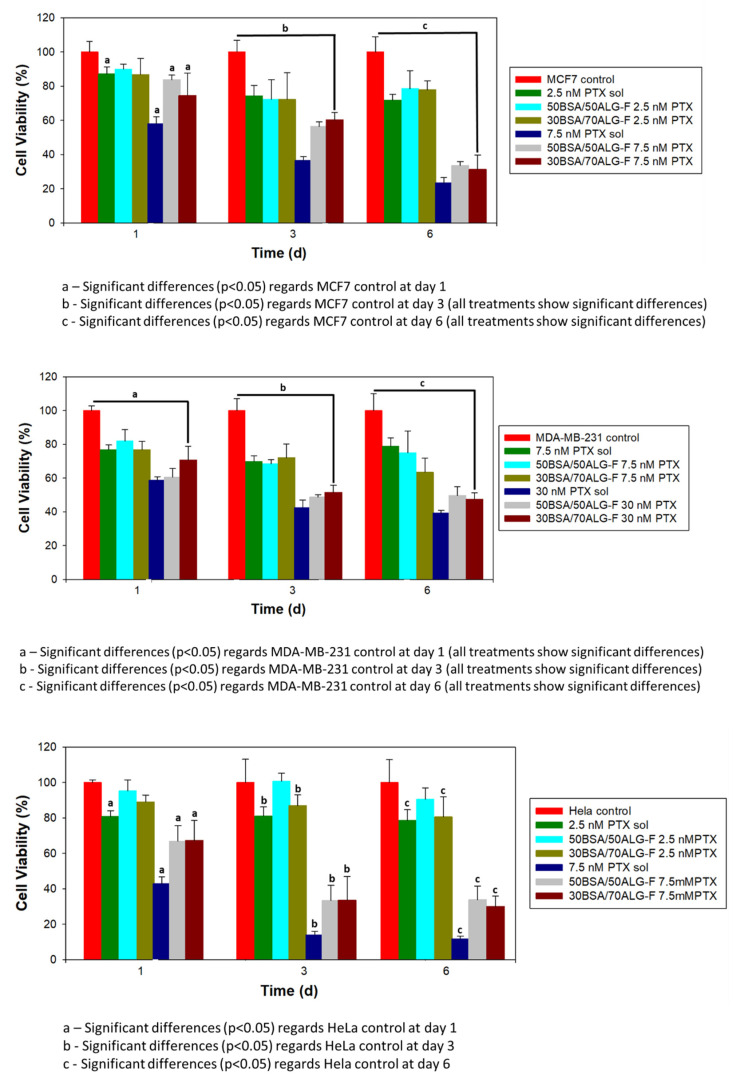
Cell viability of MCF-7, MDA-MB-231 and HeLa cells in the presence of paclitaxel (PTX) in solution and PTX-loaded nanoparticles.

PTX administered into 50BSA/50ALG–Fol and 30BSA/70ALG–Fol nanoparticles (Figure 4) caused a more gradual decrease in cell viability than PTX in solution from the first day, at the highest drug concentration assayed for MCF7, MDA-MB-231 and HeLa cells.

## 4. Discussion

Paclitaxel (PTX) is one of the most common drugs used for the treatment of different types of solid tumors [16]. PTX acts by promoting microtubule polymerization by binding to the β-subunit of tubulins, which causes mitotic arrest at the G2/M phase and results in cell death by the apoptosis pathway. Besides this, PTX exhibits cytotoxic properties that lead to the inhibition of cellular growth. Both characteristics contribute to the antitumor efficacy of PTX [29]. Since it is a hydrophobic drug, a vehicle is required to dissolve it, which causes numerous side effects, limiting its optimal clinical utility as an anticancer agent. For this reason, different nanocarriers have been prepared to improve the safety, efficacy and pharmacokinetic profile of PTX. Thus, in recent studies PTX has been encapsulated in nanoparticles [30,31], liposomes [32] and polymeric micelles [33]. On the other hand, the folate receptor is overexpressed on many tumors [34]. The FRα isoform is the most widely expressed of all the FR isoforms [35] and is overexpressed (100–300 times more than in healthy cells) in a large number of cancers of epithelial origin. Besides this, the high binding affinity (K_D_ = 0.1–1 nM) [36] of FRα for folic acid and other oxidized folates has led to the development of folate-conjugated nanosystems for targeting cancer cells [37,38]. For these reasons, folic acid was selected to target tumor cells with folate-bounded drug-loaded BSA/ALG nanoparticles.

### 4.1. Composition, Characterization and PTX Release from Folate-Conjugated Nanoparticles

Nanoparticles of different BSA/ALG composition, cross-linked with ethylenediamine (ED), were synthesized. BSA was better incorporated in 30BSA/70ALG (BSA:ALG 1:1) nanoparticles; meanwhile, alginate was similarly incorporated in all compositions, which can be attributed, at least in part, to the formation of amide bonds between ED and carboxylic groups of both ALG and BSA (each BSA molecule has 58 residues of Glu and 41 residues of Asp). TGA results confirmed the different ratio of polymers incorporated in each Np composition and could also indicate the chemical interaction between both polymers in Np.

Covalent attachment of folic acid to the three systems was achieved. There was no correlation between the amount of BSA in the nanoparticles and the extent of folate on their surface. Whereas 30BSA/70ALG–Fol and 50BSA/50ALG–Fol nanoparticles bound a similar amount of folic acid (FA), 40BSA/60ALG–Fol nanoparticles showed a significantly lower amount of FA attached. This fact could be related to the disposition of the amine groups of the side chain of amino acids in the nanoparticles; one BSA molecule has 60 residues of Lys and 26 residues of Arg, which can participate in FA attaching. Thus, the low amount of FA attached was the main reason to reject 40BSA/60ALG–Fol nanoparticles for later experiments.

TEM micrographs showed spherical, nano-sized particles without significant differences between nanoparticles of different BSA/ALG composition or those that were FA-bounded and not. The size of the unloaded nanosystems, determined by DLS (Table 2), was larger, but not statistically significantly so, for 50BSA/50ALG–Fol (268 ± 102 nm) than for 30BSA/70ALG–Fol (189 ± 81 nm), which could be due to the larger proportion of alginate in those nanoparticles, which would induce a higher swelling of the systems. However, the value of the polydispersity index was the same for both types of nanoparticles, and it was like the values obtained for other polymeric systems [12,39].

The Z-potential value of all studied nanosystems was negative (Table 2). The presence of alginate in 30BSA/70AGL and 50BSA/50ALG nanoparticles showed lightly negative Z-potential values. The FA-bounded nanosystems significantly increased Z-potential values (−69.3 mV and −66.2 mV for 30BSA/70ALG–Fol and 50BSA/50ALG–Fol, respectively), which were very much larger than −30 mV, the Z-potential value (positive or negative value) that has been established as the minimum for obtaining a physically stable nanosuspension and avoiding nanoparticle aggregation [40]. Besides this, when these nanosystems were loaded with PTX, Z-potential was of the same magnitude for PTX-loaded 30BSA/70ALG–Fol, and significantly larger (−94.1 mV) for PTX-loaded 50BSA/50ALG–Fol. This increase could be explained by the possible superficial distribution of part of the drug on the nanosystem. Thus, the low tendency to establish interactions among them, indicated by the Z-potential value of these unloaded and PTX-loaded FA-attached nanosystems, make them very suitable for later in vivo intravenous administration studies.

The PTX load of Fol-nanosystems was determined by extraction with ethanol and afterward by the enzymatic digestion of the nanoparticles with trypsin (Table 3). No differences were observed among the results obtained by both methods. Regardless of the method used, the PTX load of 50BSA/50ALG–Fol was larger (3.56–3.28 µg PTX/mg Np) than that of 30BSA/70ALG–Fol (2.63–2.42 µg PTX/mg Np). Interactions between PTX and nanosystems seem to be very similar in both types of nanoparticles since the value of the PTX load obtained by the two methods is almost the same. These observed results were in contrast with the expected results, where the drug load determined by ethanol extraction should be lower than that obtained by enzymatic digestion in the case of high PTX–nanoparticle interaction [24]. The PTX release studies showed a prolonged and faster drug release from 50BSA/50ALG–Fol nanoparticles; PTX release took place in two stages from both types of nanosystems (Figure 3). The first stage of release was during the first four hours (Table 4), and the release was faster from 50BSA/50ALG–Fol, which could be related to a part of the drug being located more superficially as indicated by the Z-potential values. A second stage of drug release was determined up to 27 h, and PTX release was faster from 50BSA/50ALG–Fol. In no case was the total amount of the PTX load released: 88% of the PTX load was released from 50BSA/50ALG–Fol and 82% of it from 30BSA/70ALG–Fol. In all these experiments, the release conditions were favored by the presence of Tween 80^®^ in the medium; however, in the absence of the surfactant, an experimental condition closer to biological systems, a slower release occurred, which would make possible a significant amount of PTX loaded into the nanoparticle–Fol to be delivered to cancer cells in vivo. Similar in vitro PTX release behavior was observed when that drug was released from nanohydrogels [27]. In that case, a maximum release took place at 50 h, and it caused a significant decrease in cell viability of human cancer cell lines. These PTX-loaded nanoparticulated systems were subcutaneously injected in female athymic nude mice bearing HeLa human tumor xenografts, and they showed higher antitumor activity than PTX in solution [41]. Equivalently, tamoxifen-loaded folate-targeted protein/polysaccharide-based nanoparticles, which released the drug in the first 8 h in in vitro studies, were demonstrated to be highly effective in tumor remission assays in MCF7 cell xenograft mice after intravenous administration [24]. In that case, although in vitro drug release from nanoparticulated and folate-targeted nanoparticulated systems took place faster, the results obtained in xenograft models demonstrated the effectiveness of these types of nanosystems as an antitumoral therapy.

In order to determine the predominant drug release mechanism from BSA/ALG–Fol nanoparticles, the obtained data were fitted to different physical models with a significant correlation coefficient (r^2^ = 0.791–0.979) (Supplementary Materials; Table S2). According to the results, the PTX release kinetics from 50BSA/50ALG–Fol nanoparticles fitted in the order of Korsemeyer–Peppas = Higuchi = First Order > Zero Order; PTX release from 30BSA/70ALG–Fol nanoparticles fitted in the order of Korsemeyer–Peppas > Higuchi > First Order > Zero Order. The Higuchi model indicates drug diffusion from the matrix, with no matrix dissolution and constant drug diffusivity [42]. The release of PTX from 30BSA/70ALG–Fol nanoparticles showed the lowest value of K_H_, which implies a slower release in comparison with 50BSA/50ALG–Fol nanoparticles. An equivalent behavior has been observed for the release of naproxen from cyclodextrin-based hydrogel matrices [43]. The release of PTX was favored by the swelling capability of the nanoparticles, which made the diffusion of the entrapped drug easier. In the case of the Korsemeyer–Peppas model, the nanosystems showed values of the release exponent (n) different from the standard value for declaring Fickian release behavior (n = 0.43; considering spherical shape) [44,45]. This was 0.36 and 0.70 for 30BSA/70ALG–Fol and 50BSA/50ALG–Fol nanoparticles, respectively. The values of the n parameter lower than 0.43 can be related to a dispersion of nanoparticle sizes. For spherical particles, when the n value is between 0.43 and 0.85, an anomalous transport (non-Fickian; intermediate between Fickian and Case II transport, zero order) is considered, and then a mixed diffusion and chain relaxation mechanism takes place, which happened for PTX release from 50BSA/50ALG–Fol. This nanoparticle composition presents an acceptable fitting to the first order kinetic model.

### 4.2. In Vitro Evaluation of Folate-Conjugated Nanoparticles in Tumor Cell Lines

The presence of FRs in several human solid tumors, such as breast cancer, has been clearly demonstrated. Thus, these receptors provide an interesting therapeutic target for solid tumor treatment. PTX is widely used as a first-line treatment for patients with breast cancer [25]. The cancer cell lines of human breast adenocarcinoma MCF-7 and MDA-MB-231 express a low and high level of folate receptor, respectively [46]. However, whereas MCF-7 cells express estrogen receptors (ER), MDA-MB-231 cells are triple negative breast cancer (TNBC) cells; they do not express ER, progesterone receptor (PR) or the amplification of HER-2/Neu [47]. Besides this, among the drugs used to treat stage IVB cervical cancer is PTX [48]. HeLa cells are human cervical carcinoma cells, positive for FR, adequate to study the efficacy of these folate-targeted PTX-loaded BSA/ALG nanocarriers.

Therefore, these three different cellular lines expressing folate receptors (FRs) were chosen for evaluating the effectiveness of these BSA/ALG–Fol nanoparticles. Different studies carried out by our group [24] and other research [49] have shown that MCF7, MDA-MB-231 and HeLa cells express different levels of FRs on their surface, meaning they can be used as an in vitro test of folate-targeted nanosystem efficacy. In general, good cytocompatibility values were obtained with the two compositions of BSA/ALG–Fol nanoparticles for the three cell lines, although a slightly lower value was observed for MDA-MB-231 cells, which can be attributed to the larger amount of nanoparticles in the experiments with this cell line. Therefore, the uptake of unloaded nanoparticle–Fol observed in these cell lines seemed not to be harmful even after six days of incubation. The presence of FRs on the cell surface led to a larger uptake in these cell lines for both nanoparticle–Fol compositions in comparison with the corresponding nanoparticle composition without folic acid bounded on their surface. A larger amount of folate receptors on the cell surface made nanoparticle–Fol uptake easier as fluorescence images of HeLa cells showed. The uptake of Nps without FA on their surface revealed there was a nonspecific uptake mechanism that would act in addition to FR-mediated internalization. In this way, different studies indicate that negative-charged nanoparticles showed high cellular uptake due to electrostatic interactions established with some parts of the cellular membrane [24,50], which would be in accordance with the negative values of the Z-potential of these BSA/ALG nanoparticles. This nonspecific uptake was lower in the case of 50BSA/50ALG nanoparticles in the three cell lines studied, and so they would be less internalized into cells without FR on their surface, like non-cancer cells.

PTX-loaded nanoparticle–Fol caused a gradual decrease in cell viability values when PTX was released from both nanosystems, mainly at a higher PTX load (7.5 nM PTX for MCF7 and HeLa; 30 nM for MDA-MB-231) at a longer time of incubation. Significant differences in decreasing viability were not observed between PTX-loaded 50BSA/50ALG–Fol and 30BSA/70ALG–Fol nanoparticles in all cell lines studied. Although viability values obtained in the presence of PTX-loaded nanoparticle–Fol were similar to those of PTX in solution, it must be taken into account that the PTX amount within the cells treated with PTX-loaded nanosystems–Fol will be lower than the amount of drug available in cells treated with PTX solutions, in accordance with in vitro drug release studies. Once PTX-loaded nanoparticle–Fol was taken up into cells via the FR-mediated endocytosis pathway, the unligate FR may then recycle to the cell surface at a rate in the range of 0.5–5 h [51]. It is believed that more nanoparticles were taken up into the cells; PTX release would have take place mainly after the enzymatic degradation of nanoparticles, which would have caused a delay in the cytotoxic action of PTX-loaded nanoparticle–Fol compared to PTX in solution.

## 5. Conclusions

The prepared folate-targeted PTX–BSA/ALG nanoparticles seem to be a promising alternative to the conventional use of Cremophor^®^ EL and would reduce the drug resistance problem mediated by P-gp. Preliminary cellular assays have demonstrated the effectiveness of these nanosystems against cancer cells overexpressing surface folate receptors. In this respect, a reduction in cell survival was observed in a cervical carcinoma cell line, a breast adenocarcinoma ER+ cell line and a TNBC cell line, which causes a very aggressive cancer type with a difficult treatment. These results allow us to think that the synthesized folate-targeted PTX–BSA/ALG nanoparticles could be considered for further in vivo biocompatibility and tumor growth suppression studies.

## Figures and Tables

**Table 1:** Nanoparticle composition. Percentage of each polymer incorporated into nanoparticles. BSA/ALG ratio in nanoparticles.

Nanoparticles	Composition of Blend (*w*/*w* Ratio)	BSA Incorporated into Nanoparticles (%)	ALG Incorporated into Nanoparticles (%)	BSA/ALG Ratio in Nanoparticles
30BSA/70ALG	2BSA:1ALG	52 ± 4	65 ± 5	1/1
40BSA/60ALG	3BSA:1ALG	13 ± 4	50 ± 2	0.25/1
50BSA/50ALG	5BSA:1ALG	25 ± 3	54 ± 4	0.5/1

**Table 2:** Mean size, polydispersity index and zeta potential of nanoparticles and folate-conjugate nanoparticles determined by quasi-elastic light scattering. Data were shown as mean ± S.D.

Nanoparticles	Mean Size (nm)	Polydispersity Index	Zeta Potential (mV)
30BSA/70ALG	182 ± 82	1.4 ± 0.7	−0.12 ± 0.04 ^a^
30BSA/70ALG–Fol	189 ± 81	1.7 ± 0.3	−69.3 ± 0.8 ^a^
PTX-loaded 30BSA/70ALG–Fol	290 ± 126	1.8 ± 0.4	−67.3 ± 0.8 ^a^
50BSA/50ALG	169 ± 28	1.5 ± 0.3	−0.43 ± 0.06 ^b^
50BSA/50ALG–Fol	268 ± 102	1.4 ± 0.3	−66.2± 0.6 ^bc^
PTX-loaded 50BSA/50ALG–Fol	296 ± 57	1.2 ± 0.4	−94.1± 0.4 ^bc^

^a^: significant statistical differences (*p* < 0.001) between 30BSA/70ALG y 30BSA/70ALG–Fol, and between 30BSA/70ALG and PTX-loaded 30BSA/70ALG–Fol; ^b^: significant statistical differences (*p* < 0.001) between 50BSA/50ALG y 50BSA/50ALG–Fol, and between 50BSA/50ALG and PTX-loaded 50BSA/50ALG–Fol; ^c^: significant statistical differences (*p* < 0.001) between 50BSA/50ALG–Fol and PTX-loaded 50BSA/50ALG–Fol.

**Table 3:** Estimation of PTX content in folate-conjugated nanoparticles (Nps–Fol).

	Drug Content in Nps–Fol	
Nanoparticles	By Extraction with Ethanol (µg PTX/mg Np)	By Tryptic Hydrolysis of the Nanoparticles (µg PTX/mg Np)
50BSA/50ALG–Fol	3.56 ± 0.13 ^a^	3.28 ± 0.24 ^b^
30BSA/70ALG–Fol	2.63 ± 0.19 ^a^	2.42 ± 0.36 ^b^

Data: mean ± S.D. (n = 3). ^a^: significant statistical differences *p* < 0.05 (*p* = 0.002); ^b^: significant statistical differences *p* < 0.05 (*p* = 0.03).

**Table 4:** Release rates of PTX from 50BSA/50ALG–Fol and 30BSA/70ALG–Fol nanoparticles.

Nanoparticles	Release Rate of PTX from Nanoparticle–Fol	
	First Stage: 0–4 h	Second Stage: 5–27 h
50BSA/50ALG–Fol	0.63 µg PTX/h per mg of Nps–Fol (r^2^ 0.973)	0.019 µg PTX/h per mg of Nps–Fol (r^2^ 0.935)
30BSA/70ALG–Fol	0.43 µg PTX/h per mg of Nps–Fol (r^2^ 0.823)	0.008 µg PTX/h per mg of Nps–Fol (r^2^ 0.877)

## Data Availability

The data presented in this study are available on request from the corresponding author.

## Supplementary Materials

The following are available online at https://www.mdpi.com/article/10.3390/polym13132083/s1, Figure S1: TGA first derivative curves of BSA, sodium alginate, 30BSA/70ALG Nps, 40BSA/60ALG Nps and 50BSA/50ALG Nps; Table S1: Percentages (*v*/*v*) of BSA (5% *w*/*v*) and ALG (1% *w*/*v*) solutions added to each blend; Table S2. Statistical parameters of nanoparticle formulations obtained after fitting the drug release data to the release kinetic models. Model fitting was attempted until 91% and 85% of drug released for PTX-30BSA/70ALG-Fol and PTX-50BSA/50ALG-Fol, respectively.
