# Supplementary material for: Paclitaxel-Loaded Folate-Targeted Albumin-Alginate Nanoparticles Crosslinked with Ethylenediamine. Synthesis and In Vitro Characterization
Source: Polymers (Basel). 2021 Jun 24;13(13):2083. doi: 10.3390/polym13132083 (PMC8272094; doi:10.3390/polym13132083)
Supplement: Supplementary file 1 [file polymers-13-02083-s001.zip › polymers-1255844-supplementary.pdf]

Supplementary material

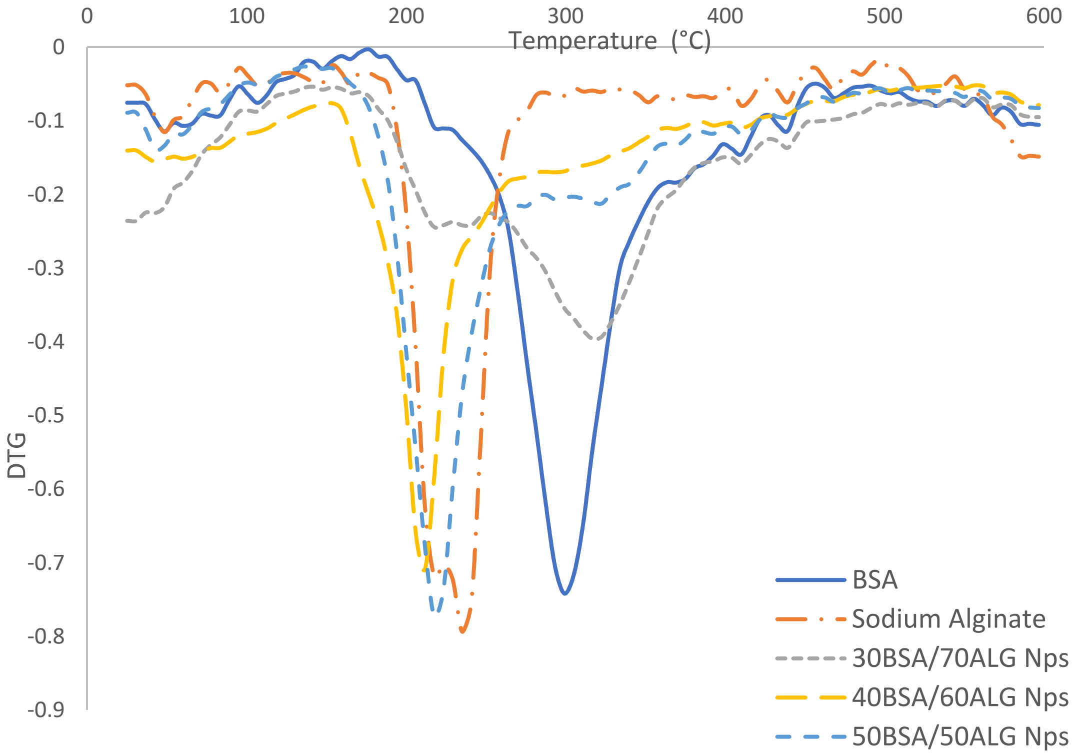

Figure S1. TGA first derivative curves of BSA, sodium alginate, 30BSA/70ALG Nps, 40BSA/60ALG Nps and 50BSA/50ALG Nps.

Table S1. Percentages (v/v) of BSA (5% w/v) and ALG (1% w/v) solutions added to each blend.

Nanoparticles	Percentage of BSA-EDAC solution added to the blend (%, v/v)	Percentage of ALG-EDAC solution added to the blend (%, v/v)	Blend composition (w/w ratio)
**30BSA/70 ALG**	30	70	2BSA:1ALG
**40BSA/60 ALG**	40	60	3BSA:1ALG
**50BSA/50 ALG**	50	50	5BSA:1ALG

Table S2 . Statistical parameters of nanoparticle formulations obtained after fitting the drug release data to the release kinetic models. Model fitting was attempted until 91% and 85% of drug released for PTX-30BSA/70ALG-Fol and PTX-50BSA/50ALG-Fol, respectively.

**NANOPARTICLES**	**HIGUCHI**	**KORSMEYER-PEPPAS**		**ZERO ORDER**	**FIRST ORDER**
	K_H $(h)^{-1/2}$	K $(h)^{-1}$	n	K_0 $(h)^{-1}$	K $(h)^{-1}$
**PTX-30BSA/70ALG-Fol**	0.892 (r^2= 0.969)	0.533	0.36 (r^2=0.979)	0.349 (r^2=0.791)	0.094 (r^2=0.882)
**PTX-50BSA/50ALG-Fol**	1.265 (r^2=0.978)	0.299	0.70 (r^2=0.979)	0.538 (r^2=0.941)	0.117 (r^2=0.979)
